# Violencia de pareja en adolescentes españoles y su relación con el apoyo familiar y social

**DOI:** 10.1016/j.aprim.2026.103492

**Published:** 2026-04-07

**Authors:** Sara Darwish-Mateos, Julia María Bohórquez-Ríos, María Angustias Becerra-Almazán, Francisco Rodríguez-Castilla, María Luz Martínez-Fernández, Alejandro Pérez-Milena

**Affiliations:** a Medicina Familiar y Comunitaria, Centro de Salud Federico del Castillo, Servicio Andaluz de Salud, Jaén, España; b Medicina Familiar y Comunitaria, Servicio de Cuidados Paliativos, Hospital Médico Quirúrgico de Jaén, Servicio Andaluz de Salud, Jaén, España; c Medicina Familiar y Comunitaria, Centro de Salud El Valle, Servicio Andaluz de Salud, Jaén, España; d Profesora de Educación Secundaria, IES Jabalcuz. Grupo de trabajo del adolescente de la Sociedad Andaluza de Medicina Familiar y Comunitaria, Jaén, España; e Medicina Familiar y Comunitaria. Centro de Salud El Valle, Servicio Andaluz de Salud. Grupo de trabajo del adolescente de la Sociedad Española de Medicina Familiar y Comunitaria. Profesor asociado de la Universidad de Jaén, Jaén, España

**Keywords:** Violencia de pareja, Conducta del adolescente, Rol de género, Estructura familiar, Intimate partner violence, Adolescent behavior, Gender rol, Family structure

## Abstract

**Objetivo:**

Conocer la prevalencia y el tipo de violencia de pareja en población adolescente y su relación con el funcionamiento familiar y el apoyo social.

**Diseño:**

Estudio descriptivo transversal.

**Emplazamiento:**

Instituto de Educación Secundaria Obligatoria.

**Participantes:**

Alumnado de 12-18 años.

**Mediciones principales:**

Encuesta autoadministrada. Cuestionario VREP (violencia ejercida, recibida y percibida), tipo de violencia (física, sexual, psicológica). Edad, sexo, estructura y función familiar (test Apgar familiar), apoyo social (cuestionario DUKE-UNC-11) y relaciones previas de pareja.

**Resultados:**

408 encuestas (tasa de respuesta 81,6%). Edad media 14,7 años[DE: 2,1], 50% mujeres. Violencia recibida: psicológica (control 55%, humillación 40,1%, social 37,9%), física (25,5%) y sexual (22,9%) (p < 0,001; χ^2^). Violencia ejercida: psicológica (control 40,4%, humillación 28,4%, social 21,8%), física (18,2%) y sexual (11,4%). La presencia de violencia es similar según sexos: los hombres realizaron más violencia física y sexual, las mujeres recibieron más violencia física (OR = 2,5) y psicológica. 80% percepción de conductas violentas, sobre todo entre quienes ejercen violencia (p < 0,10; χ^2^). Más edad estuvo asociada a recibir menos violencia social/control (OR = 0,8). Vivir en estructuras familiares no nucleares favoreció ejercer la violencia sexual (OR = 8,9), física (OR = 6,9) y psicológica social (OR = 4,9), se asoció con recibir conductas de humillación (OR = 3,6) y de control (OR = 2,5). La disfunción familiar se asoció con ejercer violencia social (OR = 2,5), más parejas previas aumentó el riesgo de recibir violencia sexual (OR = 1,3).

**Conclusiones:**

Las parejas adolescentes adoptaron y normalizaron un alto número de conductas violentas en su relación sentimental. Las mujeres, los de menos edad y la mala dinámica familiar favorecieron las conductas violentas.

## Abstract (en)

**Objective:**

To determine the prevalence and type of intimate partner violence among adolescents and its relationship with family dynamicsand social support.

**Design:**

Descriptive cross-sectional study.

**Site:**

Public urban secondary school.

**Participants:**

Students aged 12 to 18 years.

**Main measurements:**

Self-administered survey. VREP questionnaire (violence perpetrated, received, and perceived) according to the type of violence (physical, sexual, psychological). Age, sex, family structure and function (Family Apgar test), social support (DUKE-UNC-11 questionnaire), and previous intimate relationships.

**Results:**

408 surveys (response rate 81.6%). Mean age 14.7 years [SD: 2.1], 50% female. Violence received: psychological (control 55%, humiliation 40.1%, social 37.9%), physical (25.5%), and sexual (22.9%) (*P* < .001; χ^2^). Violence perpetrated: psychological (control 40.4%, humiliation 28.4%, social 21.8%), physical (18.2%) and sexual (11.4%) (*P* < .001; χ^2^). The prevalence of each type of violence was similar across genders, but men perpetrated more physical and sexual violence, and women experienced more psychological violence. 80% of participants perceived violent behavior, particularly among those who perpetrated violence (*P* < .10; χ^2^). Women experienced more physical violence (OR = 2.5). Older age was associated with experiencing less social/control violence (OR = 0.8). Living in non-nuclear family structures increased the likelihood of perpetrating sexual (OR = 8.9), physical (OR = 6.9), and social/psychological (OR = 4.9) violence, compared to experiencing humiliation (OR = 3.6) and control (OR = 2.5). Family dysfunction was associated with perpetrating social violence (OR = 2.5), while having more previous partners increased the risk of suffering sexual violence (OR = 1.3).

**Conclusions:**

Adolescent couples adopted and normalized a high number of violent behaviors in their romantic relationships. Female gender, younger age and poor family dynamics contributed to violent behaviors.

## Introducción

La violencia de pareja, también denominada violencia doméstica o violencia en el noviazgo, es un problema de salud pública asociado con numerosas consecuencias sociales, psicológicas y físicas^1^, ^2^, ^3^. Una de cada cuatro personas adolescentes ha sufrido violencia de pareja, más que la población adulta^4^, ^5^. Aunque su prevalencia ha disminuido en España en la última década^6^, ^7^, se han normalizado las conductas violentas en las parejas adolescentes, sobre todo bajo la forma de un excesivo control psicológico y social por parte de la pareja, participando tanto hombres como mujeres^2^, ^8^. Estas vivencias provocan problemas de salud mental, consumo de sustancias adictivas, riesgos en las relaciones sexuales y fracaso escolar^9^. Además, las conductas violentas en la infancia y la adolescencia generan un mayor riesgo de ser víctimas o autores de otras formas de violencia en la edad adulta^10^, ^11^. Estas conductas pueden ser precursoras de la violencia contra las mujeres en la edad adulta, favoreciendo la subordinación estructural de la mujer en todos los aspectos de la vida cotidiana^5^.

Las características de las personas adolescentes que sufren la violencia de pareja varían según las diferentes sociedades. En los países menos desarrollados influyen la tasa de escolarización y el matrimonio infantil^4^, mientras que en las sociedades industrializadas destacan la baja autoestima o el aislamiento social^7^, ^12^. En las mujeres adolescentes, la normalización de conductas violentas y el desequilibrio de poder en la relación parecen ser los determinantes más poderosos para la violencia de pareja^2^, ^6^. Otros condicionantes propuestos son el sexo, la estructura familiar, el lugar de residencia, el nivel socioeconómico o la exposición a situaciones violentas^13^, ^14^. Una buena dinámica familiar influye en el correcto crecimiento social de las personas adolescentes^15^, pero no existen evidencias firmes sobre la relación entre la vivencia de experiencias negativas en el seno familiar y la aparición de conductas violentas en la relación de pareja de los adolescentes.

El objetivo del presente estudio es conocer tanto la situación actual de la violencia de pareja en una población de adolescentes escolarizados como la relación de estas actitudes violentas con el funcionamiento familiar y el apoyo social de los menores.

## Material y métodos

Se diseñó un estudio descriptivo transversal mediante cuestionario anónimo en el primer trimestre del curso académico 2024/25. Se reclutaron adolescentes de 12 a 18 años escolarizados en un Instituto de Educación Secundaria público situado en zona urbana (Jaén, España). Los adolescentes pertenecen a familias con nivel socioeconómico y cultural medio-bajo, con un 80% de familias nucleares y sin diferencias en la tasa de fracaso escolar y absentismo respecto a otros institutos de la ciudad^15^. Se excluyeron los adolescentes con enfermedad mental grave o discapacidad intelectual. Se realizó un reclutamiento censal incluyendo la totalidad del alumnado (500 adolescentes matriculados).

Los datos se recogieron mediante la administración de un cuestionario autoadministrado, precisando 45 minutos para su cumplimentación. Un médico de familia del centro de salud de referencia fue el encargado de informar y pasar los cuestionarios al alumnado durante las horas de clase, ayudando a resolver dudas sin influir sobre las respuestas. Se aseguró el anonimato y la confidencialidad de las respuestas. La violencia de pareja se valoró con el cuestionario VREP (Violencia Ejercida, Recibida y Percibida), siempre que la relación de pareja tuviera una duración mínima de un mes. Está validado para adolescentes españoles, con buena fiabilidad psicométrica (consistencia interna adecuada: alfa de Cronbach [0,67-094]) y estabilidad temporal (correlaciones test-retest significativas)^16^. Explora la intensidad de las agresiones físicas, psicológicas y sexuales mediante un cuestionario con 28 conductas violentas clasificadas en tres categorías:

• Violencia física: conducta voluntaria que produce o puede producir daños corporales.
• Violencia sexual: obliga a un contacto sexual no deseado.
• Violencia psicológica, con tres subcategorías: aislamiento social, humillación (mediante amenazas verbales, insultos o ridiculización) y control de la pareja en sus actividades.

Se valora la conducta ejercida/recibida mediante una escala tipo Likert desde 0 (nunca) a 5 (siempre), sumándose los ítems de cada categoría. La percepción de violencia se puntúa de 1 (no es violencia) a 5 (muy violento), considerando positiva cualquier respuesta valorada con ≥ 2 puntos, dada la gravedad de los comportamientos.

Las variables independientes fueron edad, sexo, función y estructura familiar (nuclear, ampliada, monoparental o reconstituida)^17^, apoyo social, número de parejas y edad de la primera relación. El test de Apgar familiar valora la percepción del adolescente sobre la funcionalidad familiar. Es un cuestionario validado y fiable^18^, con cinco preguntas sobre la adaptabilidad, participación, crecimiento, afectividad y resolución familiar (escala Likert: 0 = «nunca/casi nunca» a 2 = «siempre/casi siempre»). Puntúa la función familiar como normal (7-10), disfunción leve (4-6) y grave (0-3). El cuestionario Duke-UNC-11 tiene validez y fiabilidad^19^ para evaluar el apoyo social, con 11 preguntas valoradas con una escala Likert (1 = «Mucho menos de lo que deseo» a 5 = «Tanto como deseo»). Puntúa apoyo social global (normal ≥ 32 puntos), confidencial (≥ 18) y afectivo (≥ 16).

Se comprobó la Normalidad de los datos (test de Kolmogorov-Smirnov) y la homocedasticidad de las varianzas (prueba de Levene) con el software SPSS v21.0©. Se hizo un estudio descriptivo obteniendo medias y proporciones, aplicando técnicas de imputación para sustituir los valores faltantes del cuestionario VREP por valores estimados según las respuestas a otros ítems de la misma categoría. Estudio bivariante según el sexo, utilizando t de Student para medias y prueba de la ji al cuadrado (χ^2^) para proporciones (α ≤ 0,05). Se calcularon regresiones logísticas binarias individuales para cada tipo de violencia de pareja, eligiendo las que presentaron un valor de p < 0,20 para realizar un modelo de regresión logística multivariante (método hacia adelante). Se comprobó la bondad de ajuste mediante la prueba de Hosmer y Lemeshow.

El estudio cuenta con la aprobación previa del Comité Ético de Investigación Científica del Complejo Hospitalario de Jaén, según RD 223/04, y autorizada por el Consejo Escolar del instituto. Se informó a padres/madres y adolescentes, entregando hoja de información y firmando consentimiento informado.

## Resultados

### Descripción de la muestra estudiada

Se recogieron un total de 408 encuestas de 500 adolescentes escolarizados (81,6% del total del alumnado). Las causas de las pérdidas fueron no acudir a clase (47%), mala cumplimentación del cuestionario (34%) y no querer participar (19%). Las principales variables estudiadas se exponen en la tabla 1, diferenciadas por sexo. La media de edad fue de 14,7 años [DE: 2,1], con igual distribución por sexo. La estructura familiar nuclear fue la más frecuente (85,3%). La puntuación media del test Apgar familiar fue 7,6 puntos [DE: 2,1] (76,4% función familiar normal). El apoyo social adecuado fue mayoritario: 88,9% apoyo social global, 88,2% confidencial y 75,9% afectivo, con una media de las puntuaciones del test Duke-UNC-11 superiores en mujeres (tabla 1, p < 0,001 test t de Student). El 44,2% de los encuestados tenían pareja en ese momento, con una media de 2,1 parejas diferentes [DE: 1,8]. La primera relación de noviazgo se estableció con una media de edad de 12,8 años [DE: 2,5].

**Tabla 1:** Características principales de los adolescentes encuestados según el sexo

	Mujeres	Hombres
*Tamaño de muestra*	208 (50,9%)	200 (49,1%)
*Edad (años)*	14,8 [DE: 2,2]	14,7 [DE: 1,9]
*Estructura familiar*		
Nuclear	88,5%	82%
Ampliada	4,3%	2%
Reconstituida	0,5%	1,5%
Monoparental	6,7%	14,5%
*Test Apgar familiar*	7,7 [DE: 1,9]	7,5 [DE: 2,4]
*Percepción de la función familiar*		
Disfunción grave	2,9%	4,5%
Disfunción leve	18,8%	21%
Normofunción	78,4%	74,5%
*Test Duke-UNC-11*		
Total (^a^)	45,8 [DE: 8,1]	41,8 [DE: 9,6]
Confidencial (^b^)	27,2 [DE: 6,1]	25,1 [DE: 6,6]
Afectivo (^c^)	18,6 [DE: 3,5]	16,7 [DE: 4,1]
*Percepción de apoyo social normal*		
Total (^d^)	94,7%	82,8%
Confidencial (^e^)	92,8%	83,4%
Afectivo (^f^)	83,7%	67,8%
*Con pareja*	41,8%	46,7%
*Edad cuando tuvo la primera pareja*	12,8 [DE: 2,7]	12,7 [DE: 2,3]
*N.° medio de parejas diferentes*	2,1 [DE: 1,7]	2,1 [DE: 1,9]

Diferencias significativas con: (^a^) p < 0,001 t de Student (t -4,48, 403 grados de libertad); (^b^) p = 0,001 t de Student (t -3,35, 404 grados de libertad); (^c^) p < 0,001 t de Student (t -4,91, 405 grados de libertad); (^d^) p < 0,001 prueba de χ^2^ (χ^2^ de Pearson 14,41; 1 grado de libertad); (^e^) p = 0,004 prueba de χ^2^ (χ^2^ de Pearson 7,48; 1 grado de libertad); (^f^) p < 0,001 prueba de χ^2^ (χ^2^ de Pearson 13,91; 1 grado de libertad).

### Resultados del cuestionario sobre violencia de pareja

**Figura 1:**
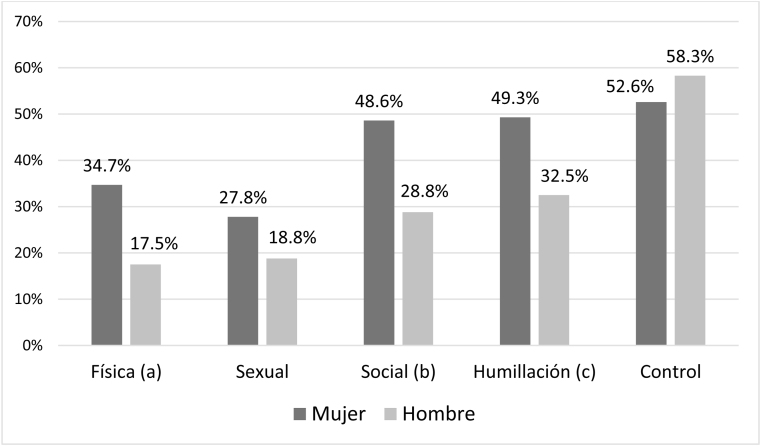
Prevalencias de violencia recibida por adolescentes valoradas con el cuestionario VERP según el sexo. Diferencias significativas con: (^a^) p = 0,015 prueba de χ^2^ (χ^2^ de Pearson 5,89; 1 grado de libertad); (^b^) p = 0,012 prueba de χ^2^ (χ^2^ de Pearson 6,34; 1 grado de libertad); (^c^) p = 0,036 prueba de χ^2^ (χ^2^ de Pearson 4,41; 1 grado de libertad)

**Figura 2:**
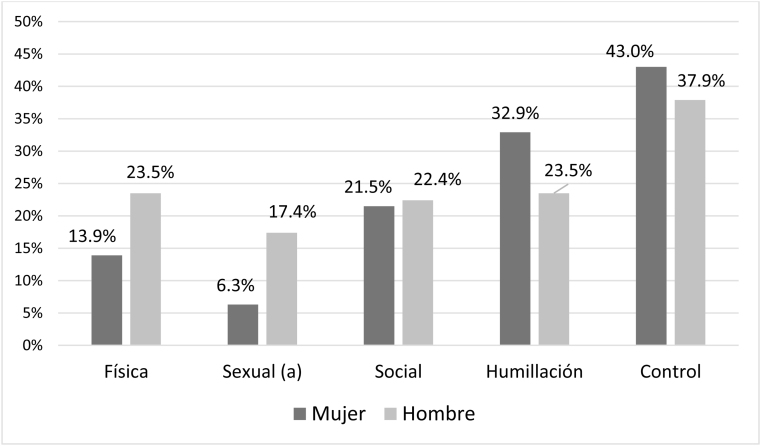
Prevalencias de violencia ejercida por adolescentes valoradas con el cuestionario VERP según el sexo. Diferencias significativas con: (^a^) p = 0,035 prueba de χ^2^ (χ^2^ de Pearson 4,43; 1 grado de libertad).

La presencia de conductas de violencia recibida fueron superiores para la violencia psicológica (social 37,9%, humillación 40,1% y control 55%) que para la física (25,5%) y la sexual (22,9%) (p < 0,001 prueba χ^2^). Las mujeres adolescentes sufrieron más violencia física (+ 17,2%), psicológica social (+ 19,8%) y de humillación (+ 16,8%) que los hombres (fig. 1; p < 0,05 prueba χ^2^). Las conductas de violencia ejercida declaradas por los adolescentes fueron similares, predominando las de tipo humillación (28,4%) y control (40,4%) frente a la psicológica social (21,8%), la física (18,2%) y la sexual (11,4%). Los hombres declararon ejercer conductas de violencia sexual en mayor porcentaje que las mujeres (+ 11,1%; p < 0,05 prueba χ^2^) (fig. 2), mientras que el resto de conductas violentas tuvieron porcentajes similares.

En la tabla 2 se detallan las conductas violentas exploradas en el cuestionario VREP y su prevalencia en función del sexo. Las mujeres adolescentes reconocían recibir todo tipo de conductas violentas (dar una bofetada, obligar a tocamientos sexuales, quedarse sin amigos o recibir gritos). El porcentaje de adolescentes que indicó ejercer violencia fue menor que quienes la recibieron. Aproximadamente el 80% de los adolescentes percibieron las diferentes conductas violentas indicadas en el cuestionario, más frecuente tanto en hombres como en las personas adolescentes que recibieron o ejercieron estas actitudes (tabla 2).

**Tabla 2:** Conductas propias de la violencia de pareja recibida, ejercida y percibida (cuestionario VREP) según el sexo de los adolescentes

	Violencia recibida		Violencia ejercida		Violencia percibida	
	Mujer	Hombre	Mujer	Hombre	Mujer	Hombre
*Violencia física*						
1. Empujar a propósito	16,2%	10%	8,9%	12,9%	66,7%	64%
6. Pegar patadas para hacer daño	9,6%	2,5%	0%	4,3%	67,1% (^a^)	82,4% (^a^)
14. Pellizcar para hacer daño	5% (^b^)	17,8% (^b^)	2,5% (^c^)	8,8% (^c^)	65,2%	78%
17. Morder o tirar del pelo adrede	16,7%	8,7%	7,6%	14,5%	66,2% (^d^)	80% (^d^)
20. Dar un tortazo o una bofetada	19,4% (^e^)	3,7% (^e^)	2,5%	7,2%	67,6% (^f^)	84% (^f^)

*Violencia sexual*						
7. Intentar mantener relaciones sexuales sin violencia física	13,7%	8,7%	0%	2,9%	67,1%	74,5%
8. Obligar a besar, aunque no apetezca	17,8%	15%	5,1%	7,2%	65,7%	64,7%
9. Negar o ridiculizar el uso de anticonceptivos	4,1%	1,2%	0%	5,8%	65,2%	72,5%
12. «No te quiero si no tienes relaciones sexuales»	9,7%	3,7%	0%	7,2%	63,8% (^g^)	78% (^g^)
21. Obligar a tocar sexualmente, aunque no apetezca	11% (^h^)	3,7% (^h^)	1,3%	5,8%	66,7%	74%
24. Obligar a mantener relaciones sexuales cuando no apetece	13,7%	5%	1,3% (^i^)	11,6% (^i^)	65,7%	78,4%

*Violencia psicológica social*						
3. Quedarse sin amigos/as	23% (^j^)	7,5% (^j^)	2,5%	7,1%	64,3%	76,9%
5. No deja ver a los/as amigos/as	6,9%	7,5%	3,8%	4,3%	62,3%	70,6%
13. No deja ver a los/as amigos/as	21,9% (^k^)	3,7% (^k^)	0%	4,3%	63,8% (^l^)	82% (^l^)
16. Insultar a la familia de la pareja	6,8%	7,5%	5,1%	6%	66,2% (^m^)	82% (^m^)
25. Insultar a los/as amigos/as de la pareja	41,1% (^n^)	22,2% (^n^)	19%	18,8%	69,6%	73,1%

*Violencia psicológica humillación*						
4. Cambiar forma de vestir, peinarse…	12,4%	3,7%	0%	2,9%	64,3%	72,5%
11. Insultar cuando se enfada	36,1%	22,2%	25,3%	21,7%	66,7%	74,5%
18. Gritar cuando se enfada	19,2% (^o^)	8,7% (^o^)	16,5%	11,6%	66,2% (^p^)	81,6% (^p^)
22. Hacer sentir que no vale para nada	12,3%	12,5%	5,1%	2,9%	69,6%	80%
26. Culpar de la violencia que se sufre	8,2%	2,5%	0%	7,2%	69,6%	78,4%
28. Poner trampas para comprobar que le quiere	20,5%	16,2%	12,7%	5,9%	66,7%	78,4%

*Violencia psicológica control*						
2. Ponerse celoso/a cuando llaman por teléfono al novio/a	29,7%	20%	7,6%	13%	62,9%	65,4%
10. Revisar objetos sin permiso	21,9%	16,2%	10,1%	9%	64,7%	72%
15. Saber en todo momento donde está la pareja	23,6%	21,5%	17,7%	14,5%	60,9% (^q^)	76% (^q^)
19. Intentar poner celoso/a al novio/a	36,1%	39,2%	27,8%	20,3%	66,2%	75,5%
23. Vigilar llamadas y redes sociales sin permiso	9,6%	5%	5,1%	2,9%	68,1%	78%
27. Acusar de coquetear con las personas que habla	23,3%	26,2%	19%	11,6%	62,9%	76,5%

Diferencias significativas con: (^a^) p = 0,061 prueba de χ^2^ (χ^2^ de Pearson 3,51; 1 grado de libertad); (^b^) p = 0,012 prueba de χ^2^ (χ^2^ de Pearson 6,34; 1 grado de libertad); (^c^) p = 0,094 prueba de χ^2^ (χ^2^ de Pearson 2,81; 1 grado de libertad); (^d^) p = 0,098 prueba de χ^2^ (χ^2^ de Pearson 2,73; 1 grado de libertad); (^e^) p = 0,002 prueba de χ^2^ (χ^2^ de Pearson 9,39; 1 grado de libertad); (^f^) p = 0,044 prueba de χ^2^ (χ^2^ de Pearson 4,06; 1 grado de libertad); (^g^) p = 0,095 prueba de χ^2^ (χ^2^ de Pearson 2,78; 1 grado de libertad); (^h^) p = 0,085 prueba de χ^2^ (χ^2^ de Pearson 2,97; 1 grado de libertad); (^i^) p = 0,009 prueba de χ^2^ (χ^2^ de Pearson 6,79; 1 grado de libertad); (^j^) p = 0,007 prueba de χ^2^ (χ^2^ de Pearson 7,24; 1 grado de libertad); (^k^) p = 0,001 prueba de χ^2^ (χ^2^ de Pearson 11,58; 1 grado de libertad); (^l^) p = 0,030 prueba de χ^2^ (χ^2^ de Pearson 4,72; 1 grado de libertad); (^m^) p = 0,056 prueba de χ^2^ (χ^2^ de Pearson 3,65; 1 grado de libertad); (^n^) p = 0,052 prueba de χ^2^ (χ^2^ de Pearson 3,79; 1 grado de libertad); (^o^) p = 0,061 prueba de χ^2^ (χ^2^ de Pearson 3,51; 1 grado de libertad); (^p^) p = 0,064 prueba de χ^2^ (χ^2^ de Pearson 3,42; 1 grado de libertad); (^q^) p = 0,083 prueba de χ^2^ (χ^2^ de Pearson 3,01; 1 grado de libertad).

### Variables relacionadas con la violencia de pareja

En el análisis bivariante, los adolescentes que ejercieron la violencia sexual y psicológica de control tenían menor edad (14,1 años [DE: 2,1]) que quienes no las realizaban (14,6 años [DE: 1,9], p < 0,05 test t de Student). La media de edad con la que tuvieron la primera pareja fue también inferior cuando habían recibido violencia psicológica social y control (12,5 años [DE: 2,3]) y cuando ejercían violencia psicológica de control (12,3 años [DE: 2,2]) (p < 0,05 test t de Student). Los adolescentes que ejercieron violencia física y sexual tuvieron un mayor número de parejas (2,3 [DE: 1,6]) que los que no la ejercían (2,8 [DE: 1,3], p < 0,05 test t de Student). Los adolescentes de familias no nucleares presentaron mayor violencia recibida, ejercida y percibida en casi todas las categorías (tabla 3). Por su parte, los adolescentes que declararon una disfunción familiar presentaron una mayor prevalencia de violencia sexual recibida y violencia social ejercida (p < 0,05 prueba de χ^2^), sufriendo con más probabilidad conductas psicológicas de humillación y de control (p < 0,10 prueba χ^2^) (tabla 3).

**Tabla 3:** Presencia de violencia recibida, percibida y ejercida según la estructura familiar del adolescente y su percepción de la función familiar empleando el test Apgar familiar

		Estructura familiar		Función familiar	
Tipo de violencia		Nuclear	No nuclear	Normal	Disfunción
Violencia física	Recibida Percibida Ejercida	21,7% (^a^) 81,1% (^b^) 18,1%	39,4% (^a^) 96,8% (^b^) 18,8%	27,2% 25% 30,6%	35,9% 30,2% 25,9%
Violencia sexual	Recibida Percibida Ejercida	17,5% (^c^) 82% (^e^) 5,2% (^f^)	42,4% (^c^) 96,8% (^e^) 33,3% (^f^)	25,4% (^d^) 26,1% 28,8%	42,9% (^d^) 30% 35,3%
Violencia psicológica: social	Recibida Percibida Ejercida	31,7% (^g^) 81,1% (^f^) 14,8% (^h^)	60,6% (^g^) 96,8% (^f^) 46,9% (^h^)	26,3% 25% 25,2% (^i^)	34,5% 69,8% 46,9% (^i^)
Violencia psicológica: humillación	Recibida Percibida Ejercida	33,3% (^j^) 82% (^l^) 25,2%	65,6% (^j^) 96,8% (^l^) 39,4%	24,2% (^k^) 26,1% 26,4%	37,7% (^k^) 30% 38,1%
Violencia psicológica: control	Recibida Percibida Ejercida	49,2% (^m^) 82% (^n^) 37,9%	75,8% (^m^) 96,8% (^n^) 50%	23,5% 26,1% 24,1% (^o^)	33,7% 30% 39% (^o^)

Diferencias significativas con: (^a^) p = 0,039 prueba de χ^2^ (χ^2^ de Pearson 4,28; 1 grado de libertad); (^b^) p = 0,033 prueba de χ^2^ (χ^2^ de Pearson 4,56; 1 grado de libertad); (^c^) p = 0,0003 prueba de χ^2^ (χ^2^ de Pearson 9,11; 1 grado de libertad); (^d^) p = 0,047 prueba de χ^2^ (χ^2^ de Pearson 3,95; 1 grado de libertad); (^e^) p = 0,039 prueba de χ^2^ (χ^2^ de Pearson 4,24; 1 grado de libertad); (^f^) p < 0,001 prueba de χ^2^ (χ^2^ de Pearson 20,15; 1 grado de libertad); (^g^) p = 0,002 prueba de χ^2^ (χ^2^ de Pearson 9,21; 1 grado de libertad); (^f^) p = 0,033 prueba de χ^2^ (χ^2^ de Pearson 4,56; 1 grado de libertad); (^h^) p< 0,001 prueba de χ^2^ (χ^2^ de Pearson 15,14; 1 grado de libertad); (^i^) p = 0,018 prueba de χ^2^ (χ^2^ de Pearson 5,60; 1 grado de libertad); (^j^) p = 0,001 prueba de χ^2^ (χ^2^ de Pearson 10,96; 1 grado de libertad); (^k^) p = 0,073 prueba de χ^2^ (χ^2^ de Pearson 3,21; 1 grado de libertad); (^l^) p = 0039, prueba de χ^2^ (χ^2^ de Pearson 4,24; 1 grado de libertad); (^m^) p = 0,007 prueba de χ^2^ (χ^2^ de Pearson 7,37; 1 grado de libertad); (^n^) p = 0,039 prueba de χ^2^ (χ^2^ de Pearson 4,24; 1 grado de libertad); (^o^) p = 0,055 prueba de χ^2^ (χ^2^ de Pearson 3,68; 1 grado de libertad).

Se realizaron análisis de regresión logística individuales y multivariantes para cada tipo de violencia, valorando su asociación con el resto de variables (edad, sexo, estructura y función familiar, apoyo social, edad de la primera relación y número de parejas). En la tabla 4 se resumen los diferentes modelos obtenidos (tablas completas en material suplementario). Las mujeres presentaron mayor riesgo de recibir violencia física (OR = 2,5). A mayor edad del adolescente, menor riesgo de sufrir violencia psicológica social (OR = 0,8) y de control (OR = 0,8), y de ejercer violencia psicológica de control (OR = 0,8). Los adolescentes con estructuras familiares no nucleares tuvieron mayor riesgo de recibir y ejercer violencia sexual (OR = 2,7/OR = 8,9), recibir violencia psicológica de humillación (OR = 3,6) y control (OR = 2,5), y de ejercer violencia psicológica social (OR = 4,9). La disfunción familiar aportó un mayor riesgo de ejercer violencia psicológica social (OR = 2,5), mientras que un mayor número de parejas previas aumentó el riesgo de recibir violencia sexual (OR = 1,3). Por último, una mayor percepción de violencia se asoció con adolescentes de mayor edad (psicológica social, OR = 7,0) y familias no nucleares (física, OR = 6,9).

**Tabla 4:** Resumen de los modelos de regresión multivariante sobre la asociación de la violencia recibida, percibida y ejercida en adolescentes con las variables independientes estudiadas (modelos completos en material suplementario)

				Intervalo de confianza al 95%			
Variable dependiente	Variable independiente	Coeficiente	OR ajustado	Inferior	Superior	χ^2^ (Wald)	p
Violencia física recibida	Sexo	0,919	2,508	1,180	5,328	5,716	0,017
Violencia física percibida	Estructura familiar	1,938	6,948	0,899	53,688	3,452	0,063
Violencia sexual recibida	Estructura familiar	0,986	2,680	1,121	6,405	4,919	0,027
	Número de parejas	0,295	1,344	1,026	1,759	4,613	0,032
Violencia sexual ejercida	Estructura familiar	2,188	8,917	2,982	26,665	15,324	< 0,001
Violencia psicológica social recibida	Edad	–0,213	0,808	0,677	0,964	5,630	0,018
	Estructura familiar	0,966	2,628	1,137	6,075	5,109	0,024
Violencia psicológica social percibida	Edad	1,938	6,948	0,899	53,688	3,452	0,063
Violencia psicológica social ejercida	Estructura familiar	1,597	4,938	2,042	11,939	12,567	< 0,001
	Función familiar	0,917	2,501	1,059	5,905	4,372	0,037
Violencia psicológica humillación recibida	Estructura familiar	1,278	3,591	1,569	8,221	9,152	0,002
Violencia psicológica control recibida	Edad	–0,230	0,795	0,666	0,948	6,554	0,010
	Estructura familiar	0,931	2,538	1,022	6,301	4,031	0,045
Violencia psicológica control ejercida	Edad	–0,224	0,800	0,673	0,950	6,464	0,011

Categorías de referencia: Sexo: (hombre = 0, mujer = 1). Estructura familiar (nuclear = 0, no nuclear = 1). Edad: (progresión de 1 año). Función familiar (normal = 0, disfunción = 1).

Modelo de regresión logística multivariante *(Forward)*. Bondad de ajuste del modelo final mediante la prueba de Hosmer y Lemeshow, con p > 0,05 en todos los casos.

## Discusión

La población adolescente estudiada declaró una importante presencia de conductas de violencia de pareja, sobre todo del tipo psicológica. Las mujeres recibieron más violencia física y psicológica (control y humillación), mientras que los hombres ejercieron más violencia física y sexual. Sin embargo, tanto hombres como mujeres adolescentes ejercieron y recibieron actos violentos, con interacciones que toleraban y normalizaban estos comportamientos. La edad, la percepción de disfunción familiar y las estructuras familiares no nucleares fueron las principales variables asociadas a conductas propias de violencia de pareja.

La prevalencia de violencia de pareja coincide con datos de otros países^1^, ^4^, ^5^, ^10^, ^20^ y de España^2^, ^6^, ^8^, ^21^, ^22^, con mayor presencia de actitudes violentas de tipo emocional y psicológica (hasta en un 25%) y menor proporción de violencia física o sexual (inferior al 10%). Las personas adolescentes implicadas en estas conductas percibieron más frecuentemente la violencia de estas actitudes, pero entendiéndolas como conductas violentas de «baja intensidad», diferentes de la violencia de género que ven en los adultos^8^, ^21^. Rechazan el sexismo hostil, pero incorporan conductas propias de un sexismo benévolo^23^, ^24^, ^25^, en gran parte influenciados por el entorno cultural^26^. Las mujeres son consideradas inocentes y afectuosas, mientras que los hombres asumen un rol de protección y responsabilidad, ejerciendo conductas violentas que subordinan a la mujer^24^. La aceptación del abuso en el noviazgo potencia los estereotipos de género, modifica la percepción de la gravedad de la violencia de pareja en la adolescencia e incrementa la frecuencia de victimización, configurando todo ello el primer paso para una escalada de violencia de pareja durante la adultez^14^, ^27^, e incluso en momentos cruciales como el embarazo^28^.

El sexo y la edad se asociaron con las conductas violentas en la relación de pareja^13^. Aunque se atribuyen diferentes tipos de conductas violentas según el sexo^8^, ^20^, ^22^, tanto hombres como mujeres adolescentes interaccionan dinámicamente como ejecutores y receptores de conductas violentas. Un estudio no encontró diferencias por sexo en la violencia cibernética en el noviazgo^29^. La madurez del adolescente se constituye como un factor protector, mientras que un mayor número de parejas sexuales se ha relacionado con una mayor propensión a conductas violentas o agresivas, en relación con la competencia sexual y otros comportamientos de riesgo^13^, ^30^.

Una adecuada dinámica familiar protege contra la violencia del noviazgo, evitando otras conductas de riesgo y mejorando el apoyo social^15^. Los adolescentes de familias no nucleares pueden haber experimentado con más frecuencia situaciones de crisis y violencia familiar que normalizarían y promoverían las actitudes violentas en la pareja^31^. Las crisis familiares no resueltas y la violencia doméstica inducirían en la adolescencia la adopción de conductas de violencia en pareja^4^, ^15^, ^22^, ^31^, ^32^. Por ello, las familias monoparentales y no nucleares precisan una atención especial que facilite la adaptación a los cambios^14^, ^33^. La cohesión familiar sería el mediador entre la estructura familiar y la salud de los adolescentes, minimizando el estrés psicosocial de los adolescentes durante los períodos de transición familiar^31^.

El estudio se ha realizado en un único instituto, por lo que habría que valorar la inferencia de resultados a zonas de alto nivel socioeconómico, rurales o adolescentes no escolarizados. Entre las limitaciones del estudio se encuentran la imposibilidad de establecer relaciones de causalidad y la dificultad para establecer una definición universal de la violencia de pareja, especialmente en la operacionalización de la agresión y las conductas violentas en el curso de una relación íntima, lo que puede dificultar la comparación de los resultados entre diferentes culturas y sociedades^14^, ^23^. La validez externa puede verse afectada por los adolescentes que no respondieron (un 18% del total) y el sesgo de deseabilidad social, que podrían infraestimar la presencia de conductas violentas.

La atención primaria desempeña un papel central para prevenir la violencia mediante la orientación comunitaria y el trabajo multidisciplinar^1^, ^3^, ^12^, ^34^. La atención integral en la adolescencia facilitaría el desarrollo de conductas saludables interpersonales y de pareja^12^. La prevención primaria promovería relaciones saludables durante la adolescencia^3^, ^34^, y la prevención secundaria evitaría posibles trayectorias hacia relaciones abusivas mediante un diagnóstico precoz^3^, ^35^.

El equipo de profesionales de atención primaria puede centrar la atención en adolescentes con familias disfuncionales y con estructuras no nucleares. Promover relaciones estables y seguras en la familia capacita al adolescente a establecer relaciones sociales no violentas^5^, ^35^. Todas estas acciones deben ser valoradas como una inversión en salud para que las personas adolescentes y los jóvenes disfruten de relaciones libres de violencia en su futuro^10^. Se precisa mayor evidencia en la eficiencia de la educación en habilidades sociales, la coordinación con el ámbito educativo, el uso de APP contra la violencia y la intervención ante la violencia de pareja establecida^34^, ^35^, ^36^, ^37^. También es preciso obtener más resultados sobre la utilidad de la atención familiar proactiva y las acciones preventivas tempranas en las escuelas^3^, ^26^, ^37^.

**Lo conocido sobre el tema**

• En la adolescencia es cada vez más frecuente la presencia de conductas violentas que afectan a las relaciones de pareja.
• Las conductas violentas suponen un desafío para la adquisición de habilidades para la resolución de conflictos entre hombres y mujeres adolescentes, y pueden desembocar en conductas de violencia de género en la adultez.

**Qué aporta este estudio**

• Hay una elevada presencia de conductas violentas en las relaciones de pareja adolescente, socialmente normalizadas, ejercidas y recibidas por los menores pese a ser percibidas como actos dañinos.
• Hay diferencias en los tipos de violencia ejercida y recibida según el sexo, existiendo otros condicionantes personales como la edad o las relaciones previas que influyen el desarrollo de violencia de pareja.
• Tanto la percepción de una mala función familiar por parte del adolescente como vivir en estructuras familiares no nucleares pueden tener una especial vulnerabilidad a ejercer o recibir actos violentos de la pareja.

## Financiación

Este trabajo se ha realizado gracias a la concesión de la beca de investigación Isabel Fernández para proyectos de investigación (referencia PI 182/21) de la Sociedad Andaluza de Medicina Familiar y Comunitaria en el año 2021, y de la ayuda económica del Distrito Sanitario Jaén - Jaén Sur del Servicio Andaluz de Salud.

## Consideraciones éticas

El estudio fue aprobado por el Comité Ético de Investigación Científica del Complejo Hospitalario de Jaén, según RD 223/04, y autorizado por el Consejo Escolar del instituto. Se informó a padres/madres y adolescentes, entregando hoja de información y firmando consentimiento informado.

## Conflicto de intereses

Los autores declaran no tener ningún conflicto de intereses.
