# Supplementary material for: Intimate partner violence among Spanish adolescents and its relationship with family and social support
Source: Aten Primaria. 2026 Apr 7;58(6):103492. [Article in Spanish] doi: 10.1016/j.aprim.2026.103492 (PMC13090547; doi:10.1016/j.aprim.2026.103492)
Supplement: Supplementary file 1 [file mmc1.doc]

**VIOLENCIA DE PAREJA EN ADOLESCENTES ESPAÑOLES Y SU RELACIÓN CON EL APOYO FAMILIAR Y SOCIAL**

**ANEXO**

**Resultados de las regresiones logísticas individuales y multivariantes para cada variable del cuestionario VREP**

Categorías de referencia:

- Sexo: (hombre=0, mujer=1);
- Edad: (progresión de 1 año);
- Estructura familiar (nuclear=0, no nuclear=1);
- Función familiar (normal=0, disfunción=1);
- Apoyo social total/confidencial/afectivo (adecuado=0, inadecuado=1).

El modelo de regresión logística multivariante f*orward* (en pasos hacia delante).

Bondad de ajuste del modelo final mediante la prueba de Hosmer y Lemeshow, con p>0,05 (en todos los modelos)

**Tabla A1. Resultado de las regresiones logísticas individuales y multivariante para la VIOLENCIA FÍSICA RECIBIDA.**

| **Resultado de las regresiones logísticas individuales** | | | | | | | | | |
| --- | --- | --- | --- | --- | --- | --- | --- | --- | --- |
| **Variable** | **n** | **B** | **E.T.** | **Wald** | **gl** | **p_valor** | **OR cruda** | **I.C. 95% para Orc** | |
|  |  |  |  |  |  |  |  | **Inferior** | **Superior** |
| Edad (numérica) | 153 | -0,051 | 0,093 | 0,297 | 1 | 0,586 | 0,951 | 0,792 | 1,140 |
| Sexo (mujer) | 152 | 0,919 | 0,385 | 5,716 | 1 | **0,017** | 2,508 | 1,180 | 5,328 |
| Estructura familiar (no nuclear) | 153 | 0,854 | 0,420 | 4,147 | 1 | **0,042** | 2,350 | 1,033 | 5,348 |
| Test Apgar familiar (puntuación numérica) | 153 | 0,006 | 0,092 | 0,004 | 1 | 0,950 | 1,006 | 0,840 | 1,204 |
| Test Apgar familiar (disfunción familiar) | 153 | 0,405 | 0,395 | 1,053 | 1 | 0,305 | 1,499 | 0,692 | 3,249 |
| Test DUKE-UNC-11 (puntuación numérica) | 152 | -0,008 | 0,023 | 0,112 | 1 | 0,738 | 0,992 | 0,948 | 1,039 |
| Test DUKE-UNC-11 (apoyo social total inadecuado) | 152 | 0,434 | 0,805 | 0,290 | 1 | 0,590 | 1,543 | 0,318 | 7,477 |
| Test DUKE-UNC-11 apoyo confidencial (puntuación numérica) | 153 | -0,009 | 0,031 | 0,081 | 1 | 0,776 | 0,991 | 0,933 | 1,053 |
| Test DUKE-UNC-11 (apoyo confidencial inadecuado) | 153 | -0,519 | 0,665 | 0,608 | 1 | 0,435 | 0,595 | 0,162 | 2,193 |
| Test DUKE-UNC-11 apoyo afectivo (puntuación numérica) | 152 | -0,030 | 0,051 | 0,355 | 1 | 0,551 | 0,970 | 0,879 | 1,071 |
| Test DUKE-UNC-11 (apoyo afectivo inadecuado) | 152 | 0,346 | 0,436 | 0,629 | 1 | 0,428 | 1,413 | 0,601 | 3,322 |
| Nº parejas previas (numérica) | 151 | 0,143 | 0,129 | 1,227 | 1 | 0,268 | 1,154 | 0,896 | 1,486 |
| Edad de inicio de la primera relación (numérica) | 151 | -0,011 | 0,074 | 0,022 | 1 | 0,882 | 0,989 | 0,855 | 1,144 |

| **Resultado de las regresiones logísticas multivariantes** | | | | | | | | | |
| --- | --- | --- | --- | --- | --- | --- | --- | --- | --- |
| **Variable** | **n** | **B** | **E.T.** | **Wald** | **gl** | **p_valor** | **OR cruda** | **I.C. 95% para Orc** | |
|  |  |  |  |  |  |  |  | **Inferior** | **Superior** |
| Sexo (mujer) | 152 | 0,919 | 0,385 | 5,716 | 1 | **0,017** | 2,508 | 1,180 | 5,328 |

**Tabla A2. Resultado de las regresiones logísticas individuales y multivariante para la VIOLENCIA FÍSICA PERCIBIDA.**

| **Resultado de las regresiones logísticas individuales** | | | | | | | | | |
| --- | --- | --- | --- | --- | --- | --- | --- | --- | --- |
| **Variable** | **n** | **B** | **E.T.** | **Wald** | **gl** | **p_valor** | **OR cruda** | **I.C. 95% para Orc** | |
|  |  |  |  |  |  |  |  | **Inferior** | **Superior** |
| Edad (numérica) | 153 | -0,113 | 0,116 | 0,949 | 1 | 0,330 | 0,893 | 0,712 | 1,121 |
| Sexo (mujer) | 152 | -0,433 | 0,456 | 0,902 | 1 | 0,342 | 0,648 | 0,265 | 1,585 |
| Estructura familiar (no nuclear) | 153 | 1,942 | 1,043 | 3,468 | 1 | 0,063 | 6,970 | 0,903 | 53,782 |
| Test Apgar familiar (puntuación numérica) | 153 | -0,081 | 0,117 | 0,479 | 1 | 0,489 | 0,922 | 0,734 | 1,159 |
| Test Apgar familiar (disfunción familiar) | 153 | 0,262 | 0,509 | 0,266 | 1 | 0,606 | 1,300 | 0,479 | 3,525 |
| Test DUKE-UNC-11 (puntuación numérica) | 152 | 0,005 | 0,027 | 0,032 | 1 | 0,858 | 1,005 | 0,952 | 1,060 |
| Test DUKE-UNC-11 (apoyo social total inadecuado) | 153 | 0,192 | 0,815 | 0,056 | 1 | 0,813 | 1,212 | 0,245 | 5,993 |
| Test DUKE-UNC-11 apoyo confidencial (puntuación numérica) | 153 | -0,009 | 0,038 | 0,052 | 1 | 0,820 | 0,991 | 0,921 | 1,068 |
| Test DUKE-UNC-11 (apoyo confidencial inadecuado) | 153 | 0,243 | 0,683 | 0,126 | 1 | 0,722 | 1,275 | 0,334 | 4,862 |
| Test DUKE-UNC-11 apoyo afectivo (puntuación numérica) | 152 | 0,045 | 0,059 | 0,563 | 1 | 0,453 | 1,046 | 0,931 | 1,174 |
| Test DUKE-UNC-11 (apoyo afectivo inadecuado) | 152 | 0,141 | 0,544 | 0,067 | 1 | 0,795 | 1,152 | 0,396 | 3,345 |
| Nº parejas previas (numérica) | 149 | 0,001 | 0,161 | 0,000 | 1 | 0,997 | 1,001 | 0,729 | 1,373 |
| Edad de inicio de la primera relación (numérica) | 149 | -0,152 | 0,103 | 2,184 | 1 | 0,139 | 0,859 | 0,703 | 1,051 |

| **Resultado de las regresiones logísticas multivariantes** | | | | | | | | | |
| --- | --- | --- | --- | --- | --- | --- | --- | --- | --- |
| **Variable** | **n** | **B** | **E.T.** | **Wald** | **gl** | **p_valor** | **OR cruda** | **I.C. 95% para Orc** | |
|  |  |  |  |  |  |  |  | **Inferior** | **Superior** |
| Estructura familiar (no nuclear) | 149 | 1,938 | 1,043 | 3,452 | 1 | 0,063 | 6,948 | 0,899 | 53,688 |

**Tabla A3. Resultado de las regresiones logísticas individuales y multivariante para la VIOLENCIA FÍSICA EJERCIDA.**

| **Resultado de las regresiones logísticas individuales** | | | | | | | | | |
| --- | --- | --- | --- | --- | --- | --- | --- | --- | --- |
| **Variable** | **n** | **B** | **E.T.** | **Wald** | **gl** | **p_valor** | **OR cruda** | **I.C. 95% para Orc** | |
|  |  |  |  |  |  |  |  | **Inferior** | **Superior** |
| Edad (numérica) | 148 | -0,030 | 0,106 | 0,079 | 1 | 0,779 | 0,971 | 0,789 | 1,194 |
| Sexo (mujer) | 147 | 0,643 | 0,433 | 2,207 | 1 | 0,137 | 1,902 | 0,814 | 4,443 |
| Estructura familiar (no nuclear) | 148 | 0,043 | 0,513 | 0,007 | 1 | 0,933 | 1,044 | 0,382 | 2,854 |
| Test Apgar familiar (puntuación numérica) | 148 | 0,133 | 0,116 | 1,315 | 1 | 0,251 | 1,142 | 0,910 | 1,434 |
| Test Apgar familiar (disfunción familiar) | 148 | -0,230 | 0,481 | 0,228 | 1 | 0,633 | 0,795 | 0,309 | 2,042 |
| Test DUKE-UNC-11 (puntuación numérica) | 148 | 0,018 | 0,028 | 0,430 | 1 | 0,512 | 1,019 | 0,964 | 1,076 |
| Test DUKE-UNC-11 (apoyo social total inadecuado) | 148 | 0,122 | 0,821 | 0,022 | 1 | 0,882 | 1,130 | 0,226 | 5,647 |
| Test DUKE-UNC-11 apoyo confidencial (puntuación numérica) | 148 | 0,036 | 0,038 | 0,896 | 1 | 0,344 | 1,036 | 0,962 | 1,116 |
| Test DUKE-UNC-11 (apoyo confidencial inadecuado) | 148 | 0,409 | 0,791 | 0,267 | 1 | 0,606 | 1,505 | 0,319 | 7,096 |
| Test DUKE-UNC-11 apoyo afectivo (puntuación numérica) | 148 | -0,006 | 0,058 | 0,010 | 1 | 0,920 | 0,994 | 0,888 | 1,113 |
| Test DUKE-UNC-11 (apoyo afectivo inadecuado) | 148 | -0,234 | 0,541 | 0,187 | 1 | 0,665 | 0,791 | 0,274 | 2,286 |
| Nº parejas previas (numérica) | 146 | 0,057 | 0,152 | 0,140 | 1 | 0,708 | 1,059 | 0,786 | 1,427 |
| Edad de inicio de la primera relación (numérica) | 146 | -0,002 | 0,086 | 0,001 | 1 | 0,982 | 0,998 | 0,843 | 1,181 |

**Tabla A4. Resultado de las regresiones logísticas individuales y multivariante para la VIOLENCIA SEXUAL RECIBIDA.**

| **Resultado de las regresiones logísticas individuales** | | | | | | | | | |
| --- | --- | --- | --- | --- | --- | --- | --- | --- | --- |
| **Variable** | **n** | **B** | **E.T.** | **Wald** | **gl** | **p_valor** | **OR cruda** | **I.C. 95% para Orc** | |
|  |  |  |  |  |  |  |  | **Inferior** | **Superior** |
| Edad (numérica) | 153 | 0,026 | 0,097 | 0,070 | 1 | 0,791 | 1,026 | 0,849 | 1,240 |
| Sexo (mujer) | 152 | 0,511 | 0,389 | 1,725 | 1 | 0,189 | 1,667 | 0,778 | 3,572 |
| Estructura familiar (no nuclear) | 153 | 1,245 | 0,426 | 8,530 | 1 | **0,003** | 3,474 | 1,506 | 8,011 |
| Test Apgar familiar (puntuación numérica) | 153 | -0,096 | 0,092 | 1,077 | 1 | 0,299 | 0,909 | 0,759 | 1,089 |
| Test Apgar familiar (disfunción familiar) | 153 | 0,788 | 0,402 | 3,853 | 1 | **0,050** | 2,200 | 1,001 | 4,834 |
| Test DUKE-UNC-11 (puntuación numérica) | 152 | 0,000 | 0,024 | 0,000 | 1 | 0,985 | 1,000 | 0,954 | 1,050 |
| Test DUKE-UNC-11 apoyo confidencial (puntuación numérica) | 153 | 0,000 | 0,032 | 0,000 | 1 | 0,995 | 1,000 | 0,939 | 1,066 |
| Test DUKE-UNC-11 (apoyo confidencial inadecuado) | 153 | 0,362 | 0,668 | 0,294 | 1 | 0,588 | 1,436 | 0,388 | 5,313 |
| Test DUKE-UNC-11 apoyo afectivo (puntuación numérica) | 152 | -0,017 | 0,053 | 0,109 | 1 | 0,742 | 0,983 | 0,887 | 1,090 |
| Test DUKE-UNC-11 (apoyo afectivo inadecuado) | 152 | -0,086 | 0,479 | 0,032 | 1 | 0,857 | 0,917 | 0,359 | 2,345 |
| Nº parejas previas (numérica) | 151 | 0,367 | 0,134 | 7,478 | 1 | **0,006** | 1,443 | 1,109 | 1,876 |
| Edad de inicio de la primera relación (numérica) | 151 | -0,037 | 0,075 | 0,235 | 1 | 0,628 | 0,964 | 0,832 | 1,118 |

| **Resultado de las regresiones logísticas multivariantes** | | | | | | | | | |
| --- | --- | --- | --- | --- | --- | --- | --- | --- | --- |
| **Variable** | **n** | **B** | **E.T.** | **Wald** | **gl** | **p_valor** | **OR cruda** | **I.C. 95% para Orc** | |
|  |  |  |  |  |  |  |  | **Inferior** | **Superior** |
| Estructura familiar (no nuclear) | 150 | 0,986 | 0,445 | 4,919 | 1 | **0,027** | 2,680 | 1,121 | 6,405 |
| Nº parejas previas (numérica) | 150 | 0,295 | 0,137 | 4,613 | 1 | **0,032** | 1,344 | 1,026 | 1,759 |

**Tabla A5. Resultado de las regresiones logísticas individuales y multivariante para la VIOLENCIA SEXUAL PERCIBIDA.**

| **Resultado de las regresiones logísticas individuales** | | | | | | | | | |
| --- | --- | --- | --- | --- | --- | --- | --- | --- | --- |
| **Variable** | **n** | **B** | **E.T.** | **Wald** | **gl** | **p_valor** | **OR cruda** | **I.C. 95% para Orc** | |
|  |  |  |  |  |  |  |  | **Inferior** | **Superior** |
| Edad (numérica) | 153 | -0,078 | 0,116 | 0,445 | 1 | 0,505 | 0,925 | 0,737 | 1,162 |
| Sexo (mujer) | 152 | -0,553 | 0,468 | 1,395 | 1 | 0,238 | 0,575 | 0,230 | 1,440 |
| Estructura familiar (no nuclear) | 153 | 1,887 | 1,043 | 3,271 | 1 | 0,071 | 6,600 | 0,854 | 51,019 |
| Test Apgar familiar (puntuación numérica) | 153 | -0,071 | 0,118 | 0,361 | 1 | 0,548 | 0,932 | 0,739 | 1,174 |
| Test Apgar familiar (disfunción familiar) | 153 | 0,194 | 0,512 | 0,144 | 1 | 0,705 | 1,214 | 0,445 | 3,312 |
| Test DUKE-UNC-11 (puntuación numérica) | 153 | 0,005 | 0,028 | 0,034 | 1 | 0,853 | 1,005 | 0,952 | 1,062 |
| Test DUKE-UNC-11 (apoyo social total inadecuado) | 153 | 0,247 | 0,817 | 0,092 | 1 | 0,762 | 1,280 | 0,258 | 6,346 |
| Test DUKE-UNC-11 apoyo confidencial (puntuación numérica) | 153 | -0,012 | 0,039 | 0,091 | 1 | 0,763 | 0,988 | 0,916 | 1,066 |
| Test DUKE-UNC-11 (apoyo confidencial inadecuado) | 153 | 0,300 | 0,685 | 0,192 | 1 | 0,661 | 1,350 | 0,353 | 5,166 |
| Test DUKE-UNC-11 apoyo afectivo (puntuación numérica) | 152 | 0,053 | 0,060 | 0,775 | 1 | 0,379 | 1,055 | 0,937 | 1,187 |
| Test DUKE-UNC-11 (apoyo afectivo inadecuado) | 153 | 0,077 | 0,547 | 0,020 | 1 | 0,888 | 1,080 | 0,370 | 3,153 |
| Nº parejas previas (numérica) | 149 | -0,030 | 0,160 | 0,035 | 1 | 0,852 | 0,970 | 0,709 | 1,329 |
| Edad de inicio de la primera relación (numérica) | 149 | -0,124 | 0,102 | 1,483 | 1 | 0,223 | 0,883 | 0,723 | 1,079 |

**Tabla A6. Resultado de las regresiones logísticas individuales y multivariante para la VIOLENCIA SEXUAL EJERCIDA.**

| **Resultado de las regresiones logísticas individuales** | | | | | | | | | |
| --- | --- | --- | --- | --- | --- | --- | --- | --- | --- |
| **Variable** | **n** | **B** | **E.T.** | **Wald** | **gl** | **p_valor** | **OR cruda** | **I.C. 95% para Orc** | |
|  |  |  |  |  |  |  |  | **Inferior** | **Superior** |
| Edad (numérica) | 149 | -0,278 | 0,135 | 4,252 | 1 | **0,039** | 0,758 | 0,582 | 0,986 |
| Sexo (mujer) | 148 | 1,136 | 0,561 | 4,108 | 1 | **0,043** | 3,116 | 1,038 | 9,351 |
| Estructura familiar (no nuclear) | 149 | 2,216 | 0,559 | 15,727 | 1 | **<0,001** | 9,167 | 3,067 | 27,401 |
| Test Apgar familiar (puntuación numérica) | 149 | -0,090 | 0,123 | 0,537 | 1 | 0,464 | 0,914 | 0,719 | 1,162 |
| Test Apgar familiar (disfunción familiar) | 149 | 0,300 | 0,543 | 0,305 | 1 | 0,581 | 1,349 | 0,466 | 3,909 |
| Test DUKE-UNC-11 (puntuación numérica) | 148 | -0,014 | 0,033 | 0,183 | 1 | 0,669 | 0,986 | 0,925 | 1,051 |
| Test DUKE-UNC-11 (apoyo social total inadecuado) | 149 | -0,015 | 0,043 | 0,116 | 1 | 0,733 | 0,985 | 0,905 | 1,073 |
| Test DUKE-UNC-11 apoyo confidencial (puntuación numérica) | 149 | 0,718 | 1,067 | 0,454 | 1 | 0,501 | 2,051 | 0,254 | 16,594 |
| Test DUKE-UNC-11 (apoyo confidencial inadecuado) | 148 | -0,065 | 0,071 | 0,841 | 1 | 0,359 | 0,937 | 0,816 | 1,076 |
| Test DUKE-UNC-11 apoyo afectivo (puntuación numérica) | 148 | 0,570 | 0,581 | 0,962 | 1 | 0,327 | 1,768 | 0,566 | 5,519 |
| Test DUKE-UNC-11 (apoyo afectivo inadecuado) | 149 | -0,015 | 0,043 | 0,116 | 1 | 0,733 | 0,985 | 0,905 | 1,073 |
| Nº parejas previas (numérica) | 147 | 0,287 | 0,159 | 3,251 | 1 | 0,071 | 1,332 | 0,975 | 1,820 |
| Edad de inicio de la primera relación (numérica) | 147 | -0,095 | 0,094 | 1,020 | 1 | 0,313 | 0,909 | 0,756 | 1,094 |

| **Resultado de las regresiones logísticas multivariantes** | | | | | | | | | |
| --- | --- | --- | --- | --- | --- | --- | --- | --- | --- |
| **Variable** | **n** | **B** | **E.T.** | **Wald** | **gl** | **p_valor** | **OR cruda** | **I.C. 95% para Orc** | |
|  |  |  |  |  |  |  |  | **Inferior** | **Superior** |
| Estructura familiar (no nuclear) | 146 | 2,188 | 0,559 | 15,324 | 1 | **<0,001** | 8,917 | 2,982 | 26,665 |

**Tabla A7. Resultado de las regresiones logísticas individuales y multivariante para la VIOLENCIA PSICOLÓGICA SOCIAL RECIBIDA.**

| **Resultado de las regresiones logísticas individuales** | | | | | | | | | |
| --- | --- | --- | --- | --- | --- | --- | --- | --- | --- |
| **Variable** | **n** | **B** | **E.T.** | **Wald** | **gl** | **p_valor** | **OR cruda** | **I.C. 95% para Orc** | |
|  |  |  |  |  |  |  |  | **Inferior** | **Superior** |
| Edad (numérica) | 153 | -0,249 | 0,087 | 8,148 | 1 | **0,004** | 0,780 | 0,657 | 0,925 |
| Sexo (mujer) | 152 | 0,852 | 0,341 | 6,224 | 1 | **0,013** | 2,344 | 1,200 | 4,578 |
| Estructura familiar (no nuclear) | 153 | 1,200 | 0,407 | 8,703 | 1 | **0,003** | 3,320 | 1,496 | 7,368 |
| Test Apgar familiar (puntuación numérica) | 153 | -0,182 | 0,084 | 4,724 | 1 | **0,030** | 0,833 | 0,707 | 0,982 |
| Test Apgar familiar (disfunción familiar) | 153 | 0,388 | 0,361 | 1,151 | 1 | 0,283 | 1,474 | 0,726 | 2,992 |
| Test DUKE-UNC-11 (puntuación numérica) | 152 | -0,023 | 0,021 | 1,200 | 1 | 0,273 | 0,977 | 0,938 | 1,018 |
| Test DUKE-UNC-11 (apoyo social total inadecuado) | 152 | 0,504 | 0,699 | 0,520 | 1 | 0,471 | 1,655 | 0,421 | 6,511 |
| Test DUKE-UNC-11 apoyo confidencial (puntuación numérica) | 153 | -0,043 | 0,028 | 2,326 | 1 | 0,127 | 0,958 | 0,906 | 1,012 |
| Test DUKE-UNC-11 (apoyo confidencial inadecuado) | 153 | 0,427 | 0,561 | 0,580 | 1 | 0,446 | 1,533 | 0,511 | 4,598 |
| Test DUKE-UNC-11 apoyo afectivo (puntuación numérica) | 152 | -0,006 | 0,045 | 0,020 | 1 | 0,887 | 0,994 | 0,909 | 1,086 |
| Test DUKE-UNC-11 (apoyo afectivo inadecuado) | 153 | -0,231 | 0,414 | 0,311 | 1 | 0,577 | 0,793 | 0,352 | 1,788 |
| Nº parejas previas (numérica) | 151 | 0,187 | 0,122 | 2,362 | 1 | 0,124 | 1,206 | 0,950 | 1,531 |
| Edad de inicio de la primera relación (numérica) | 149 | -0,113 | 0,068 | 2,706 | 1 | 0,100 | 0,894 | 0,781 | 1,022 |

| **Resultado de las regresiones logísticas multivariantes** | | | | | | | | | |
| --- | --- | --- | --- | --- | --- | --- | --- | --- | --- |
| **Variable** | **n** | **B** | **E.T.** | **Wald** | **gl** | **p_valor** | **OR cruda** | **I.C. 95% para Orc** | |
|  |  |  |  |  |  |  |  | **Inferior** | **Superior** |
| Edad (numérica) | 149 | -0,213 | 0,090 | 5,630 | 1 | **0,018** | 0,808 | 0,677 | 0,964 |
| Estructura familiar (no nuclear) | 149 | 0,966 | 0,427 | 5,109 | 1 | **0,024** | 2,628 | 1,137 | 6,075 |

**Tabla A8. Resultado de las regresiones logísticas individuales y multivariante para la VIOLENCIA PSICOLÓGICA SOCIAL PERCIBIDA.**

| **Resultado de las regresiones logísticas individuales** | | | | | | | | | |
| --- | --- | --- | --- | --- | --- | --- | --- | --- | --- |
| **Variable** | **n** | **B** | **E.T.** | **Wald** | **gl** | **p_valor** | **OR cruda** | **I.C. 95% para Orc** | |
|  |  |  |  |  |  |  |  | **Inferior** | **Superior** |
| Edad (numérica) | 153 | -0,060 | 0,114 | 0,279 | 1 | 0,597 | 0,942 | 0,754 | 1,177 |
| Sexo (mujer) | 152 | -0,433 | 0,456 | 0,902 | 1 | 0,342 | 0,648 | 0,265 | 1,585 |
| Estructura familiar (no nuclear) | 153 | 1,942 | 1,043 | 3,468 | 1 | 0,063 | 6,970 | 0,903 | 53,782 |
| Test Apgar familiar (puntuación numérica) | 153 | -0,095 | 0,118 | 0,643 | 1 | 0,423 | 0,910 | 0,722 | 1,146 |
| Test Apgar familiar (disfunción familiar) | 153 | 0,262 | 0,509 | 0,266 | 1 | 0,606 | 1,300 | 0,479 | 3,525 |
| Test DUKE-UNC-11 (puntuación numérica) | 153 | -0,002 | 0,028 | 0,005 | 1 | 0,945 | 0,998 | 0,945 | 1,054 |
| Test DUKE-UNC-11 (apoyo social total inadecuado) | 152 | 0,192 | 0,815 | 0,056 | 1 | 0,813 | 1,212 | 0,245 | 5,993 |
| Test DUKE-UNC-11 apoyo confidencial (puntuación numérica) | 153 | -0,023 | 0,038 | 0,362 | 1 | 0,547 | 0,977 | 0,906 | 1,054 |
| Test DUKE-UNC-11 (apoyo confidencial inadecuado) | 153 | 0,243 | 0,683 | 0,126 | 1 | 0,722 | 1,275 | 0,334 | 4,862 |
| Test DUKE-UNC-11 apoyo afectivo (puntuación numérica) | 153 | 0,048 | 0,059 | 0,656 | 1 | 0,418 | 1,049 | 0,934 | 1,179 |
| Test DUKE-UNC-11 (apoyo afectivo inadecuado) | 153 | 0,141 | 0,544 | 0,067 | 1 | 0,795 | 1,152 | 0,396 | 3,345 |
| Nº parejas previas (numérica) | 149 | 0,001 | 0,161 | 0,000 | 1 | 0,997 | 1,001 | 0,729 | 1,373 |
| Edad de inicio de la primera relación (numérica) | 149 | -0,131 | 0,101 | 1,687 | 1 | 0,194 | 0,877 | 0,720 | 1,069 |

| **Resultado de las regresiones logísticas multivariantes** | | | | | | | | | |
| --- | --- | --- | --- | --- | --- | --- | --- | --- | --- |
| **Variable** | **n** | **B** | **E.T.** | **Wald** | **gl** | **p_valor** | **OR cruda** | **I.C. 95% para Orc** | |
|  |  |  |  |  |  |  |  | **Inferior** | **Superior** |
| Estructura familiar (no nuclear) | 149 | 1,938 | 1,043 | 3,452 | 1 | 0,063 | 6,948 | 0,899 | 53,688 |

**Tabla A9. Resultado de las regresiones logísticas individuales y multivariante para la VIOLENCIA PSICOLÓGICA SOCIAL EJERCIDA.**

| **Resultado de las regresiones logísticas individuales** | | | | | | | | | |
| --- | --- | --- | --- | --- | --- | --- | --- | --- | --- |
| **Variable** | **n** | **B** | **E.T.** | **Wald** | **gl** | **p_valor** | **OR cruda** | **I.C. 95% para Orc** | |
|  |  |  |  |  |  |  |  | **Inferior** | **Superior** |
| Edad (numérica) | 147 | 0,021 | 0,100 | 0,043 | 1 | 0,836 | 1,021 | 0,840 | 1,241 |
| Sexo (mujer) | 146 | 0,051 | 0,401 | 0,016 | 1 | 0,899 | 1,052 | 0,479 | 2,309 |
| Estructura familiar (no nuclear) | 147 | 1,627 | 0,441 | 13,602 | 1 | **<0,001** | 5,087 | 2,143 | 12,074 |
| Test Apgar familiar (puntuación numérica) | 147 | -0,202 | 0,097 | 4,306 | 1 | **0,038** | 0,817 | 0,676 | 0,989 |
| Test Apgar familiar (disfunción familiar) | 147 | 0,962 | 0,414 | 5,392 | 1 | **0,020** | 2,617 | 1,162 | 5,893 |
| Test DUKE-UNC-11 (puntuación numérica) | 147 | -0,236 | 0,505 | 0,218 | 1 | 0,640 | 0,790 | 0,294 | 2,125 |
| Test DUKE-UNC-11 apoyo confidencial (puntuación numérica) | 147 | -0,021 | 0,034 | 0,395 | 1 | 0,530 | 0,979 | 0,916 | 1,046 |
| Test DUKE-UNC-11 (apoyo confidencial inadecuado) | 147 | 1,458 | 1,055 | 1,909 | 1 | 0,167 | 4,297 | 0,543 | 33,995 |
| Test DUKE-UNC-11 apoyo afectivo (puntuación numérica) | 147 | 0,006 | 0,055 | 0,010 | 1 | 0,919 | 1,006 | 0,903 | 1,119 |
| Test DUKE-UNC-11 (apoyo afectivo inadecuado) | 147 | -0,236 | 0,505 | 0,218 | 1 | 0,640 | 0,790 | 0,294 | 2,125 |
| Nº parejas previas (numérica) | 145 | 0,026 | 0,146 | 0,033 | 1 | 0,856 | 1,027 | 0,772 | 1,366 |
| Edad de inicio de la primera relación (numérica) | 145 | 0,072 | 0,086 | 0,702 | 1 | 0,402 | 1,075 | 0,908 | 1,274 |

| **Resultado de las regresiones logísticas multivariantes** | | | | | | | | | |
| --- | --- | --- | --- | --- | --- | --- | --- | --- | --- |
| **Variable** | **n** | **B** | **E.T.** | **Wald** | **gl** | **p_valor** | **OR cruda** | **I.C. 95% para Orc** | |
|  |  |  |  |  |  |  |  | **Inferior** | **Superior** |
| Test Apgar familiar (disfunción familiar) | 147 | 0,917 | 0,438 | 4,372 | 1 | **0,037** | 2,501 | 1,059 | 5,905 |

**Tabla A10. Resultado de las regresiones logísticas individuales y multivariante para la VIOLENCIA PSICOLÓGICA HUMILLACIÓN RECIBIDA.**

| **Resultado de las regresiones logísticas individuales** | | | | | | | | | |
| --- | --- | --- | --- | --- | --- | --- | --- | --- | --- |
| **Variable** | **n** | **B** | **E.T.** | **Wald** | **gl** | **p_valor** | **OR cruda** | **I.C. 95% para Orc** | |
|  |  |  |  |  |  |  |  | **Inferior** | **Superior** |
| Edad (numérica) | 152 | -0,136 | 0,084 | 2,604 | 1 | 0,107 | 0,873 | 0,740 | 1,030 |
| Sexo (mujer) | 151 | 0,703 | 0,337 | 4,357 | 1 | **0,037** | 2,019 | 1,044 | 3,906 |
| Estructura familiar (no nuclear) | 152 | 1,340 | 0,420 | 10,197 | 1 | **0,001** | 3,818 | 1,678 | 8,689 |
| Test Apgar familiar (puntuación numérica) | 152 | -0,100 | 0,081 | 1,517 | 1 | 0,218 | 0,904 | 0,771 | 1,061 |
| Test Apgar familiar (disfunción familiar) | 152 | 0,641 | 0,360 | 3,167 | 1 | 0,075 | 1,898 | 0,937 | 3,846 |
| Test DUKE-UNC-11 (puntuación numérica) | 151 | -0,030 | 0,021 | 2,045 | 1 | 0,153 | 0,970 | 0,931 | 1,011 |
| Test DUKE-UNC-11 (apoyo social total inadecuado) | 151 | -0,253 | 0,630 | 0,161 | 1 | 0,688 | 0,776 | 0,226 | 2,668 |
| Test DUKE-UNC-11 apoyo confidencial (puntuación numérica) | 152 | -0,052 | 0,028 | 3,415 | 1 | 0,065 | 0,949 | 0,898 | 1,003 |
| Test DUKE-UNC-11 (apoyo confidencial inadecuado) | 152 | -0,049 | 0,523 | 0,009 | 1 | 0,926 | 0,952 | 0,342 | 2,655 |
| Test DUKE-UNC-11 apoyo afectivo (puntuación numérica) | 151 | -0,015 | 0,045 | 0,115 | 1 | 0,734 | 0,985 | 0,902 | 1,076 |
| Test DUKE-UNC-11 (apoyo afectivo inadecuado) | 151 | 0,143 | 0,400 | 0,127 | 1 | 0,721 | 1,153 | 0,527 | 2,524 |
| Nº parejas previas (numérica) | 150 | 0,038 | 0,120 | 0,099 | 1 | 0,754 | 1,038 | 0,821 | 1,314 |
| Edad de inicio de la primera relación (numérica) | 150 | -0,045 | 0,067 | 0,464 | 1 | 0,496 | 0,956 | 0,838 | 1,089 |

| **Resultado de las regresiones logísticas multivariantes** | | | | | | | | | |
| --- | --- | --- | --- | --- | --- | --- | --- | --- | --- |
| **Variable** | **n** | **B** | **E.T.** | **Wald** | **gl** | **p_valor** | **OR cruda** | **I.C. 95% para Orc** | |
|  |  |  |  |  |  |  |  | **Inferior** | **Superior** |
| Estructura familiar (no nuclear) | 150 | 1,278 | 0,423 | 9,152 | 1 | **0,002** | 3,591 | 1,569 | 8,221 |

**Tabla A11. Resultado de las regresiones logísticas individuales y multivariante para la VIOLENCIA PSICOLÓGICA HUMILLACIÓN PERCIBIDA.**

| **Resultado de las regresiones logísticas individuales** | | | | | | | | | |
| --- | --- | --- | --- | --- | --- | --- | --- | --- | --- |
| **Variable** | **n** | **B** | **E.T.** | **Wald** | **gl** | **p_valor** | **OR cruda** | **I.C. 95% para Orc** | |
|  |  |  |  |  |  |  |  | **Inferior** | **Superior** |
| Edad (numérica) | 153 | -0,078 | 0,116 | 0,445 | 1 | 0,505 | 0,925 | 0,737 | 1,162 |
| Sexo (mujer) | 152 | -0,553 | 0,468 | 1,395 | 1 | 0,238 | 0,575 | 0,230 | 1,440 |
| Estructura familiar (no nuclear) | 153 | 1,887 | 1,043 | 3,271 | 1 | 0,071 | 6,600 | 0,854 | 51,019 |
| Test Apgar familiar (puntuación numérica) | 153 | -0,071 | 0,118 | 0,361 | 1 | 0,548 | 0,932 | 0,739 | 1,174 |
| Test Apgar familiar (disfunción familiar) | 153 | 0,194 | 0,512 | 0,144 | 1 | 0,705 | 1,214 | 0,445 | 3,312 |
| Test DUKE-UNC-11 (puntuación numérica) | 153 | 0,005 | 0,028 | 0,034 | 1 | 0,853 | 1,005 | 0,952 | 1,062 |
| Test DUKE-UNC-11 (apoyo social total inadecuado) | 153 | 0,247 | 0,817 | 0,092 | 1 | 0,762 | 1,280 | 0,258 | 6,346 |
| Test DUKE-UNC-11 apoyo confidencial (puntuación numérica) | 153 | -0,012 | 0,039 | 0,091 | 1 | 0,763 | 0,988 | 0,916 | 1,066 |
| Test DUKE-UNC-11 (apoyo confidencial inadecuado) | 153 | 0,300 | 0,685 | 0,192 | 1 | 0,661 | 1,350 | 0,353 | 5,166 |
| Test DUKE-UNC-11 apoyo afectivo (puntuación numérica) | 153 | 0,053 | 0,060 | 0,775 | 1 | 0,379 | 1,055 | 0,937 | 1,187 |
| Test DUKE-UNC-11 (apoyo afectivo inadecuado) | 153 | 0,077 | 0,547 | 0,020 | 1 | 0,888 | 1,080 | 0,370 | 3,153 |
| Nº parejas previas (numérica) | 149 | -0,030 | 0,160 | 0,035 | 1 | 0,852 | 0,970 | 0,709 | 1,329 |
| Edad de inicio de la primera relación (numérica) | 149 | -0,124 | 0,102 | 1,483 | 1 | 0,223 | 0,883 | 0,723 | 1,079 |

**Tabla A12. Resultado de las regresiones logísticas individuales y multivariante para la VIOLENCIA PSICOLÓGICA HUMILLACIÓN EJERCIDA.**

| **Resultado de las regresiones logísticas individuales** | | | | | | | | | |
| --- | --- | --- | --- | --- | --- | --- | --- | --- | --- |
| **Variable** | **n** | **B** | **E.T.** | **Wald** | **gl** | **p_valor** | **OR cruda** | **I.C. 95% para Orc** | |
|  |  |  |  |  |  |  |  | **Inferior** | **Superior** |
| Edad (numérica) | 148 | -0,053 | 0,090 | 0,346 | 1 | 0,557 | 0,948 | 0,795 | 1,131 |
| Sexo (mujer) | 147 | -0,466 | 0,373 | 1,565 | 1 | 0,211 | 0,627 | 0,302 | 1,303 |
| Estructura familiar (no nuclear) | 148 | 0,656 | 0,416 | 2,489 | 1 | 0,115 | 1,928 | 0,853 | 4,356 |
| Test Apgar familiar (puntuación numérica) | 148 | -0,096 | 0,089 | 1,170 | 1 | 0,279 | 0,908 | 0,763 | 1,081 |
| Test Apgar familiar (disfunción familiar) | 148 | 0,539 | 0,387 | 1,943 | 1 | 0,163 | 1,714 | 0,803 | 3,658 |
| Test DUKE-UNC-11 (puntuación numérica) | 147 | 0,002 | 0,024 | 0,004 | 1 | 0,947 | 1,002 | 0,956 | 1,049 |
| Test DUKE-UNC-11 apoyo confidencial (puntuación numérica) | 148 | -0,028 | 0,031 | 0,781 | 1 | 0,377 | 0,973 | 0,915 | 1,034 |
| Test DUKE-UNC-11 (apoyo confidencial inadecuado) | 148 | 0,095 | 0,615 | 0,024 | 1 | 0,877 | 1,100 | 0,330 | 3,669 |
| Test DUKE-UNC-11 apoyo afectivo (puntuación numérica) | 147 | 0,063 | 0,051 | 1,495 | 1 | 0,221 | 1,065 | 0,963 | 1,178 |
| Test DUKE-UNC-11 (apoyo afectivo inadecuado) | 147 | -0,588 | 0,498 | 1,397 | 1 | 0,237 | 0,555 | 0,209 | 1,473 |
| Nº parejas previas (numérica) | 146 | -0,093 | 0,142 | 0,427 | 1 | 0,514 | 0,911 | 0,690 | 1,204 |
| Edad de inicio de la primera relación (numérica) | 146 | -0,040 | 0,072 | 0,312 | 1 | 0,576 | 0,961 | 0,835 | 1,106 |

**Tabla A13. Resultado de las regresiones logísticas individuales y multivariante para la VIOLENCIA PSICOLÓGICA CONTROL RECIBIDA.**

| **Resultado de las regresiones logísticas individuales** | | | | | | | | | |
| --- | --- | --- | --- | --- | --- | --- | --- | --- | --- |
| **Variable** | **n** | **B** | **E.T.** | **Wald** | **gl** | **p_valor** | **OR cruda** | **I.C. 95% para Orc** | |
|  |  |  |  |  |  |  |  | **Inferior** | **Superior** |
| Edad (numérica) | 151 | -0,251 | 0,086 | 8,437 | 1 | **0,004** | 0,778 | 0,657 | 0,922 |
| Sexo (mujer) | 150 | 0,234 | 0,329 | 0,504 | 1 | 0,478 | 1,263 | 0,662 | 2,410 |
| Estructura familiar (no nuclear) | 151 | 1,173 | 0,446 | 6,921 | 1 | **0,009** | 3,233 | 1,349 | 7,748 |
| Test Apgar familiar (puntuación numérica) | 151 | -0,138 | 0,084 | 2,695 | 1 | 0,101 | 0,871 | 0,739 | 1,027 |
| Test Apgar familiar (disfunción familiar) | 151 | 0,504 | 0,368 | 1,869 | 1 | 0,172 | 1,655 | 0,804 | 3,405 |
| Test DUKE-UNC-11 (puntuación numérica) | 150 | -0,019 | 0,021 | 0,805 | 1 | 0,370 | 0,981 | 0,942 | 1,022 |
| Test DUKE-UNC-11 (apoyo social total inadecuado) | 150 | 0,805 | 0,650 | 1,537 | 1 | 0,215 | 2,238 | 0,626 | 7,995 |
| Test DUKE-UNC-11 apoyo confidencial (puntuación numérica) | 151 | -0,038 | 0,028 | 1,843 | 1 | 0,175 | 0,963 | 0,911 | 1,017 |
| Test DUKE-UNC-11 (apoyo confidencial inadecuado) | 151 | 0,358 | 0,516 | 0,480 | 1 | 0,488 | 1,430 | 0,520 | 3,933 |
| Test DUKE-UNC-11 apoyo afectivo (puntuación numérica) | 150 | 0,004 | 0,045 | 0,008 | 1 | 0,930 | 1,004 | 0,920 | 1,095 |
| Test DUKE-UNC-11 (apoyo afectivo inadecuado) | 150 | -0,238 | 0,399 | 0,357 | 1 | 0,550 | 0,788 | 0,360 | 1,723 |
| Nº parejas previas (numérica) | 149 | 0,088 | 0,122 | 0,517 | 1 | 0,472 | 1,092 | 0,860 | 1,386 |
| Edad de inicio de la primera relación (numérica) | 149 | -0,146 | 0,072 | 4,142 | 1 | **0,042** | 0,864 | 0,751 | 0,995 |

| **Resultado de las regresiones logísticas multivariantes** | | | | | | | | | |
| --- | --- | --- | --- | --- | --- | --- | --- | --- | --- |
| **Variable** | **n** | **B** | **E.T.** | **Wald** | **gl** | **p_valor** | **OR cruda** | **I.C. 95% para Orc** | |
|  |  |  |  |  |  |  |  | **Inferior** | **Superior** |
| Edad (numérica) | 149 | -0,230 | 0,090 | 6,554 | 1 | **0,010** | 0,795 | 0,666 | 0,948 |
| Estructura familiar (no nuclear) | 149 | 0,931 | 0,464 | 4,031 | 1 | **0,045** | 2,538 | 1,022 | 6,301 |

**Tabla A14. Resultado de las regresiones logísticas individuales y multivariante para la VIOLENCIA PSICOLÓGICA CONTROL PERCIBIDA.**

| **Resultado de las regresiones logísticas individuales** | | | | | | | | | |
| --- | --- | --- | --- | --- | --- | --- | --- | --- | --- |
| **Variable** | **n** | **B** | **E.T.** | **Wald** | **gl** | **p_valor** | **OR cruda** | **I.C. 95% para Orc** | |
|  |  |  |  |  |  |  |  | **Inferior** | **Superior** |
| Edad (numérica) | 153 | -0,078 | 0,116 | 0,445 | 1 | 0,505 | 0,925 | 0,737 | 1,162 |
| Sexo (mujer) | 152 | -0,553 | 0,468 | 1,395 | 1 | 0,238 | 0,575 | 0,230 | 1,440 |
| Estructura familiar (no nuclear) | 153 | 1,887 | 1,043 | 3,271 | 1 | 0,071 | 6,600 | 0,854 | 51,019 |
| Test Apgar familiar (puntuación numérica) | 153 | -0,071 | 0,118 | 0,361 | 1 | 0,548 | 0,932 | 0,739 | 1,174 |
| Test Apgar familiar (disfunción familiar) | 153 | 0,194 | 0,512 | 0,144 | 1 | 0,705 | 1,214 | 0,445 | 3,312 |
| Test DUKE-UNC-11 (puntuación numérica) | 153 | 0,005 | 0,028 | 0,034 | 1 | 0,853 | 1,005 | 0,952 | 1,062 |
| Test DUKE-UNC-11 (apoyo social total inadecuado) | 153 | 0,247 | 0,817 | 0,092 | 1 | 0,762 | 1,280 | 0,258 | 6,346 |
| Test DUKE-UNC-11 apoyo confidencial (puntuación numérica) | 153 | -0,012 | 0,039 | 0,091 | 1 | 0,763 | 0,988 | 0,916 | 1,066 |
| Test DUKE-UNC-11 (apoyo confidencial inadecuado) | 153 | 0,300 | 0,685 | 0,192 | 1 | 0,661 | 1,350 | 0,353 | 5,166 |
| Test DUKE-UNC-11 apoyo afectivo (puntuación numérica) | 153 | 0,053 | 0,060 | 0,775 | 1 | 0,379 | 1,055 | 0,937 | 1,187 |
| Test DUKE-UNC-11 (apoyo afectivo inadecuado) | 153 | 0,077 | 0,547 | 0,020 | 1 | 0,888 | 1,080 | 0,370 | 3,153 |
| Nº parejas previas (numérica) | 149 | -0,030 | 0,160 | 0,035 | 1 | 0,852 | 0,970 | 0,709 | 1,329 |
| Edad de inicio de la primera relación (numérica) | 149 | -0,124 | 0,102 | 1,483 | 1 | 0,223 | 0,883 | 0,723 | 1,079 |

**Tabla A15. Resultado de las regresiones logísticas individuales y multivariante para la VIOLENCIA PSICOLÓGICA CONTROL EJERCIDA.**

| **Resultado de las regresiones logísticas individuales** | | | | | | | | | |
| --- | --- | --- | --- | --- | --- | --- | --- | --- | --- |
| **Variable** | **n** | **B** | **E.T.** | **Wald** | **gl** | **p_valor** | **OR cruda** | **I.C. 95% para Orc** | |
|  |  |  |  |  |  |  |  | **Inferior** | **Superior** |
| Edad (numérica) | 146 | -0,220 | 0,087 | 6,321 | 1 | **0,012** | 0,803 | 0,676 | 0,953 |
| Sexo (mujer) | 145 | -0,214 | 0,341 | 0,396 | 1 | 0,529 | 0,807 | 0,414 | 1,573 |
| Estructura familiar (no nuclear) | 146 | 0,492 | 0,412 | 1,427 | 1 | 0,232 | 1,636 | 0,729 | 3,671 |
| Test Apgar familiar (puntuación numérica) | 146 | -0,066 | 0,084 | 0,608 | 1 | 0,436 | 0,936 | 0,794 | 1,105 |
| Test Apgar familiar (disfunción familiar) | 146 | 0,697 | 0,366 | 3,626 | 1 | 0,057 | 2,008 | 0,980 | 4,115 |
| Test DUKE-UNC-11 (puntuación numérica) | 146 | -0,006 | 0,021 | 0,091 | 1 | 0,763 | 0,994 | 0,953 | 1,036 |
| Test DUKE-UNC-11 (apoyo social total inadecuado) | 146 | 0,491 | 0,712 | 0,475 | 1 | 0,491 | 1,633 | 0,405 | 6,590 |
| Test DUKE-UNC-11 apoyo confidencial (puntuación numérica) | 146 | -0,017 | 0,029 | 0,346 | 1 | 0,556 | 0,983 | 0,930 | 1,040 |
| Test DUKE-UNC-11 (apoyo confidencial inadecuado) | 146 | 0,338 | 0,576 | 0,345 | 1 | 0,557 | 1,403 | 0,454 | 4,335 |
| Test DUKE-UNC-11 apoyo afectivo (puntuación numérica) | 146 | 0,013 | 0,046 | 0,082 | 1 | 0,775 | 1,013 | 0,927 | 1,108 |
| Test DUKE-UNC-11 (apoyo afectivo inadecuado) | 146 | -0,156 | 0,412 | 0,144 | 1 | 0,704 | 0,855 | 0,382 | 1,917 |
| Nº parejas previas (numérica) | 144 | -0,086 | 0,127 | 0,456 | 1 | 0,500 | 0,918 | 0,715 | 1,178 |
| Edad de inicio de la primera relación (numérica) | 144 | -0,136 | 0,071 | 3,699 | 1 | 0,054 | 0,873 | 0,760 | 1,003 |

| **Resultado de las regresiones logísticas multivariantes** | | | | | | | | | |
| --- | --- | --- | --- | --- | --- | --- | --- | --- | --- |
| **Variable** | **n** | **B** | **E.T.** | **Wald** | **gl** | **p_valor** | **OR cruda** | **I.C. 95% para Orc** | |
|  |  |  |  |  |  |  |  | **Inferior** | **Superior** |
| Edad (numérica) | 144 | -0,224 | 0,088 | 6,464 | 1 | **0,011** | 0,800 | 0,673 | 0,950 |
